# Exploring telediagnostic procedures in child neuropsychiatry: addressing ADHD diagnosis and autism symptoms through supervised machine learning

**DOI:** 10.1007/s00787-023-02145-4

**Published:** 2023-01-25

**Authors:** Silvia Grazioli, Alessandro Crippa, Eleonora Rosi, Antonio Candelieri, Silvia Busti Ceccarelli, Maddalena Mauri, Martina Manzoni, Valentina Mauri, Sara Trabattoni, Massimo Molteni, Paola Colombo, Maria Nobile

**Affiliations:** 1 Child Psychopathology Unit, Scientific Institute, IRCCS Eugenio Medea, Via Don Luigi Monza, 20, Bosisio Parini, Lecco, Italy; 2 Department of Economics, Management and Statistics, University of Milano-Bicocca, Milan, Italy; 3 PhD School in Neuroscience, School of Medicine and Surgery, University of Milano-Bicocca, Milan, Italy

**Keywords:** Telehealth, Diagnostic process, Attention deficit/hyperactivity disorder, Machine learning, Autism spectrum disorders

## Abstract

Recently, there has been an increase in telemedicine applied to child neuropsychiatry, such as the use of online platforms to collect remotely case histories and demographic and behavioral information. In the present proof-of-concept study, we aimed to understand to what extent information parents and teachers provide through online questionnaires overlaps with clinicians’ diagnostic conclusions on attention-deficit/hyperactivity disorder (ADHD). Moreover, we intended to explore a possible role that autism spectrum disorders (ASD) symptoms played in this process. We examined parent- and teacher-rated questionnaires collected remotely and an on-site evaluation of intelligence quotients from 342 subjects (18% females), aged 3–16 years, and referred for suspected ADHD. An easily interpretable machine learning model—decision tree (DT)—was built to simulate the clinical process of classifying ADHD/non-ADHD based on collected data. Then, we tested the DT model’s predictive accuracy through a cross-validation approach. The DT classifier’s performance was compared with those that other machine learning models achieved, such as random forest and support vector machines. Differences in ASD symptoms in the DT-identified classes were tested to address their role in performing a diagnostic error using the DT model. The DT identified the decision rules clinicians adopt to classify an ADHD diagnosis with an 82% accuracy rate. Regarding the cross-validation experiment, our DT model reached a predictive accuracy of 74% that was similar to those of other classification algorithms. The caregiver-reported ADHD core symptom severity proved the most discriminative information for clinicians during the diagnostic decision process. However, ASD symptoms were a confounding factor when ADHD severity had to be established. Telehealth procedures proved effective in obtaining an automated output regarding a diagnostic risk, reducing the time delay between symptom detection and diagnosis. However, this should not be considered an alternative to on-site procedures but rather as automated support for clinical practice, enabling clinicians to allocate further resources to the most complex cases.

**Supplementary Information:**

The online version contains supplementary material available at 10.1007/s00787-023-02145-4.

## Introduction

Over the 10 past years, healthcare services have been involved in a progressive digitalization process [1]. The COVID-19 pandemic spurred this trend, increasing the demand for effective telehealth support for mental health [2]. Accordingly, the development and use of online platforms for collecting case history, demographic, and behavioral data are steadily increasing in child and adolescent neuropsychiatry [3, 4]. However, the validity and reliability of data collected remotely via computer are still to be ascertained [5]. In fact, although clinical questionnaires already are being delivered through apps on smart devices, the uncontrolled settings of administration might affect the validity of self-reported data, which could differ from the original settings of the validated questionnaires [6]. Moreover, remote self-administration prevents users from seeking a clinician for help in properly understanding an item’s content.

Our proof-of-concept study addresses this topic regarding a diagnosis of attention deficit / hyperactivity disorder (ADHD) because the evaluation process for this condition reflects the trend toward digitalization described above. According to the National Institute for Health and Care Excellence Guidelines [7], an accurate ADHD diagnostic process requires integrating different instruments and informants.

Within this workflow, ADHD characteristics are investigated—to a certain degree—through parent and teacher reports that could be digitally administered. A recent study demonstrated that parents and teachers showed similar diagnostic accuracy in predicting a clinical diagnosis when considering the ADHD Rating Scale-IV threshold to discriminate ADHD/non-ADHD condition [8]. However, parents with lower educational attainment showed worse diagnostic accuracy when compared both to parents with higher education levels and to teachers [8]. Remote collection of behavioral data could potentially enhance this effect because individuals with lower educational levels may face difficulties accessing digital tools [9].

The present study has two objectives. First, we aimed to understand to what extent expert clinicians’ diagnostic conclusions overlap with the information parents and teachers provide via online questionnaires. To do this, we tested a decision tree (DT) classification, which is an interpretable machine learning (ML) algorithm, to analyze diagnostic data collected at the Scientific Institute “IRCCS Eugenio Medea” Regional Center for ADHD [10]. Here, we recently developed the first Italian web-based screening tool to administer remotely digital clinical questionnaires to provide timely and effective support for the diagnostic process in the child neuropsychiatry field [11]. Recent evidence has shown the advantages of ML algorithms as well-suited analytic techniques for digitally obtained diagnostic data within the progressive digitalization process under way in clinical practice over the past years [12]. Computer algorithms can be optimized to highlight patterns in remotely collected clinical data that could assign a predicted diagnostic label to each evaluated subject. At this stage, most of the studies employing ML techniques to support the ADHD diagnostic process models have provided accurate but not easily interpretable results [13, 14]. However, in the specific case of supporting decisions associated with a diagnosis, model interpretability is crucially important in enabling clinicians to integrate qualitative clinical knowledge with algorithms’ results. In addition, our DT classification’s predictive performance was tested through a cross-validation approach to simulate predictions in new help-seeking subjects. We examined the reproducibility of the prediction results through direct comparisons with different ML models, such as a random forest (RF) and a support vector machine (SVM).

Second, we intended to explore whether caregivers reported a co-presence of autism spectrum disorder (ASD) symptoms. We aimed to understand at which point the presence of autistic features in children clinically referred for ADHD problems could represent a potential confounding factor, also taking into account the considerable behavioral overlap between the two disorders [15, 16].

## Methods

In this retrospective, single-center, observational study, we reported data from the diagnostic process of a sample of children and adolescents referred for suspected ADHD diagnosis at the Scientific Institute “IRCCS Eugenio Medea”—Associazione La Nostra Famiglia in Bosisio Parini (Lecco, Italy)—between early 2017 and late 2020. The Institute’s Ethical Review Board (Prot. N. 29/22, “Comitato Etico IRCCS E. Medea—Sezione Scientifica Associazione La Nostra Famiglia”) approved this study and all the participants’ legal guardians gave their written informed consent to the children’s participation.

### Participants

Participants included 342 children and adolescents (18% females) living in Northern Italy, aged 3 to 16 years, who underwent a full neuropsychiatric evaluation and did or did not receive an ADHD and/or ASD diagnosis in accordance with the *DSM-5* criteria [17].

### Procedure

**Fig. 1:**
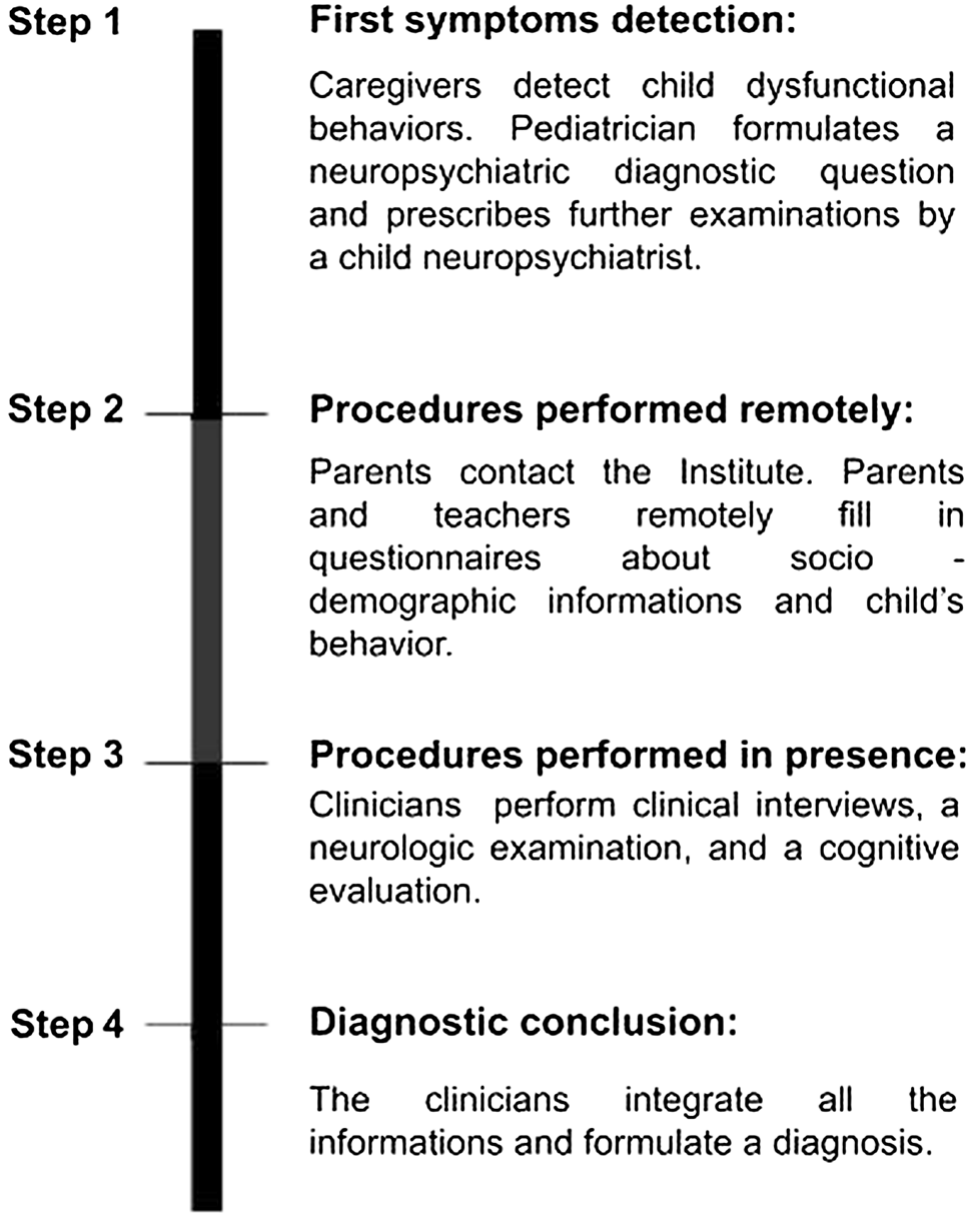
Diagnostic process scheme. Graphic design of diagnostic process at the Regional Center for ADHD

A workflow of the diagnostic procedure is shown in Fig. 1.

### Measures

#### Remotely collected measures

*Case history and demographic questionnaire.* The following information was collected: (a) age and sex; (b) perinatal risk factors, including pregnancy problems, preterm or late birth, extremely high or low birth weight, breastfeeding problems, and APGAR score at birth; (c) family type (biological/fostering parents, co-parenting/single-parent); and (d) family socioeconomic status coded according to the Hollingshead scale [18].

*Parent-report questionnaires*. Conners' Parent Rating Scale–Revised (CPRS-R) [19]. The CPRS-R is appropriate for parents with children between the ages of 3 and 17. It consists of items addressing behavioral and emotional problems. Item scores are summed up in symptom scales, presenting moderate to high internal reliability, with Cronbach’s alpha ranging from 0.75 to 0.94 [19]. CPRS-R adjusted scores higher than 60 and 70 indicate moderate and severe clinical risk. For this questionnaire, the factors considered were: oppositional, cognitive problems, hyperactivity, anxious/shy, perfectionism, social problems, psychosomatic problems, ADHD Index, CGI Restless-Impulsive, CGI Emotional Lability, CGI Total, DSM-IV Inattentive, DSM-IV Hyperactive-Impulsive, and DSM-IV Combined.

Child Behavior Checklist (CBCL) for Ages 1.5–5 or CBCL for Ages 6–18 [20, 21]. The CBCL is a questionnaire assessing behavioral and emotional problems in children and adolescents, covering a broad spectrum of psychopathological symptoms. The intraclass correlation coefficient of CBCL is 0.95; Cronbach’s alpha ranges from 0.72 to 0.97 [20, 21]. Symptom scale scores higher than 64 and 69 indicate moderate and severe risk, respectively. Scores higher than 59 and 63 on Total Problems, Internalizing Problems, and Externalizing Problems scales indicate moderate and severe risk, respectively. In our analyses, we included the following scale scores: Anxious/Depressed, Withdrawn/Depressed, Somatic Complaints, Attention Problems, Rule-Breaking Behavior, Aggressive Behavior, Depressive Problems, Anxiety Problems, ADHD Problems, Oppositional Defiant Problems, Internalizing Problems, Externalizing Problems, and Total Problems.

Social Responsiveness Scale (SRS) [22]. The SRS is a questionnaire collecting information on the impairment severity of numerous social abilities linked to ASD symptoms in children and adolescents. SRS is characterized by high internal consistency (Cronbach’s alpha ranging from 0.94 to 0.96) [23]. Scores higher than 59 and 76 indicate moderate and severe risk, respectively. SRS showed a sensitivity and a specificity value of 0.92 [23]. Social Awareness, Social Cognition, Social Communication, Social Motivation, Autistic Mannerisms (Restricted Interests and Repetitive Behavior), and Total Scores were considered in the analyses.

*Teacher-report questionnaire*. Conners’ Teacher Rating Scale–Revised (CTRS-R) [24]. The CTRS-R measures behavioral problems in children and adolescents aged 3–17 years. The CTRS-R showed Cronbach’s alpha coefficients higher than 0.73. Scores higher than 60 and 70 indicate moderate and severe risk, respectively. We considered for the analyses the same scales as for the parent version, except for Psychosomatic Problems (not included in the teacher form).

#### Measures administered on-site

A medical doctor specialized in child neuropsychiatry fully examined all participants. A child psychologist with experience in ADHD and ASD independently confirmed the diagnosis via direct observation of the child and administered neuropsychological and cognitive tests.

*Intelligence quotient evaluation*. The child’s IQ was evaluated through one of the following scales according to age, testability, and ability: Griffiths Mental Development Scales [25]; Wechsler Preschool and Primary Scale of Intelligence, Third Edition [26]; and Wechsler Intelligence Scale for Children, Fourth Edition [27].

### Statistical analyses

Statistical analyses were performed using R version 4.1.2 [28].

#### DT model simulating the clinical process of classifying ADHD/non-ADHD on collected data

To ascertain which reported features held the most relevance in the diagnostic process of ADHD, we used a DT classifier, which is a flowchart-like structure that is built considering the full data set “sitting” at the top of the root node, and at each junction, observations satisfying the splitting condition are assigned to the left branch and the others to the right branch [29]. Information gain is used as a node impurity measure to select the attribute and split each node until reaching the last node, the so-called “leaf” [30]. The most frequently observed class in each leaf is considered as a classification prediction by the algorithm [31].

Note that:

- *TP* means *true positive*: the subjects who the clinicians diagnosed with ADHD and who the DT correctly classified as “ADHD”*;*
- *TN* means *true negative*: the subjects who the clinicians did not diagnose with ADHD and who the DT correctly classified “non-ADHD”*;*
- *FP* means *false positive*: the subjects who the clinicians did not diagnose with ADHD and who the DT wrongly classified as “ADHD”;
- *FN* means *false negative*: the subjects who the clinicians diagnosed with ADHD and who the DT wrongly classified as “non-ADHD*.*”

The algorithm performance was evaluated considering the following information [32].

- *Classification accuracy*: percentage of correctly performed predictions against the total number of instances.
- *No information rate (NIR)*: the accuracy achievable by always predicting the majority class label.
- *P-Value of Acc* > *NIR*: a hypothesis test result to evaluate whether the algorithm’s classification accuracy is greater than the rate of the largest class (NIR).
- *Specificity*: percentage of correctly performed negative predictions (non-ADHD) against the number of subjects without an ADHD diagnosis.
- *Sensitivity*: percentage of correctly performed positive predictions (ADHD) against the number of subjects with an ADHD diagnosis.
- *Positive predictive value (PPV)*: percentage of subjects with an ADHD diagnosis against the number of all positive predictions (ADHD).
- *Negative predictive value (NPV)*: percentage of subjects without an ADHD diagnosis against the number of all negative predictions (non-ADHD).

#### Cross-validation experiment of the DT model and its predictive accuracy in comparison to other ML models

To evaluate the generalization performances of each ML-trained model, a leave-one-out (LOO) cross-validation technique was used because it provides an accurate estimate of the probability of error [33]. Furthermore, to check for the robustness of DT-based results, two complementary ML models were tested [33]: random forest (RF) and support vector machine (SVM). RF is an ensemble learning technique that generates many DTs and aggregates their results after performing a bootstrap of the sample’s subjects and randomly selecting a subset of the predictors [34]. In contrast, the SVM method identifies an optimal hyperplane to separate correctly two classes of subjects through the maximization of the distance between observed data points [35].

#### Identification of DT rules for correct/incorrect classifications

Classifications of ADHD/non-ADHD diagnoses the DT algorithm performed were compared with the clinicians’ actual ADHD/non-ADHD diagnoses. To address the clinical characteristics of subjects the DT algorithm incorrectly classified (in other words, the cases in which the integration of in-person observation, caregiver questionnaires, and psychometric tests the clinicians administered was discordant with the data resulting exclusively from questionnaires), the whole data set was split into ADHD and non-ADHD children. Correct/incorrect classification was the dependent variable in two further ML models to identify a rule-based algorithm that could express the properties of misclassified subjects. ML analyses were performed as previously described.

Moreover, correct/incorrect classification was an independent variable and socio-demographic and cognitive information were dependent variables in non-parametric analyses to address whether these factors could be associated with the DT model’s performance.

#### Analysis of autism symptoms in correctly/incorrectly classified ADHD children

To disentangle the role of ASD symptoms in the DT algorithm’s correct/incorrect classification, subjects with or without an ADHD diagnosis were considered separately in two contingency tables addressing frequencies of correctly/incorrectly classified ADHD by the DT and the presence versus absence of an ASD diagnosis as the clinicians assessed. Two Fisher’s exact tests were applied to test the association between receiving an ASD diagnosis and being correctly or incorrectly classified as ADHD through the DT algorithm.

In addition, parent-reported ASD symptoms were evaluated regarding the correctly classified/misclassified ADHD subjects. TP/TN/FP/FN categories independent variables and the six SRS scores were dependent variables in separate Kruskal–Wallis tests. To identify what specific couples of medians were significantly different, two-sided pairwise Wilcoxon Rank Sum tests with Bonferroni correction for multiple comparisons were performed. The following group comparisons were considered: (a) TP versus FN and (b) TN versus FP.

## Results

After performing data cleaning procedures (see the Supplementary Information section), more than 50% of data were missing for 16 subjects, which we therefore excluded from the analyses. The final sample consisted of 326 children and adolescents. Table 1 shows the participants’ characteristics. The male-to-female ratio (5.5:1) in our sample is in line with previous literature [36]. At the end of the clinicians’ diagnostic process, 52% of the sample received an ADHD diagnosis without ASD, 33% of the subjects received neither an ADHD diagnosis nor an ASD diagnosis, 8% of the subjects were diagnosed with ASD without ADHD, and 7% of the children received a comorbid ADHD–ASD diagnosis.

**Table 1:** Sample descriptive statistics

Variable	Total sample	ADHD stratification		ASD stratification	
		ADHD	Non—ADHD	ASD	Non-ASD
Age	9 ($\pm$ 2)	9 ($\pm$ 2)	8 ($\pm$ 2)	8 ($\pm$ 2)	9 ($\pm$ 2)
Sex	m = 83% f = 17%	m = 89% f = 11%	m = 75% f = 25%	m = 86% f = 14%	m = 83% f = 17%
N. of Perinatal Problems	1 ($\pm$ 1)	1 ($\pm$ 1)	1 ($\pm$ 1)	1 ($\pm$ 1)	1 ($\pm$ 1)
SES	50 ($\pm$ 18)	50 ($\pm$ 17)	50 ($\pm$ 19)	50 ($\pm$ 19)	50 ($\pm$ 18)
IQ	96 ($\pm$ 16)	96 ($\pm$ 15)	96 ($\pm$ 17)	97 ($\pm$ 16)	96 ($\pm$ 16)

The location parameter for quantitative variables is the median ($\pm$ standard deviation). *ADHD *Attention deficit/hyperactivity disorder, *ASD *Autism spectrum disorder, *f *females, *IQ *Intelligence quotient,* m* males, *SES *Socio-economic status

### DT model simulating the clinical process of classifying ADHD/non-ADHD on collected data

**Fig. 2:**
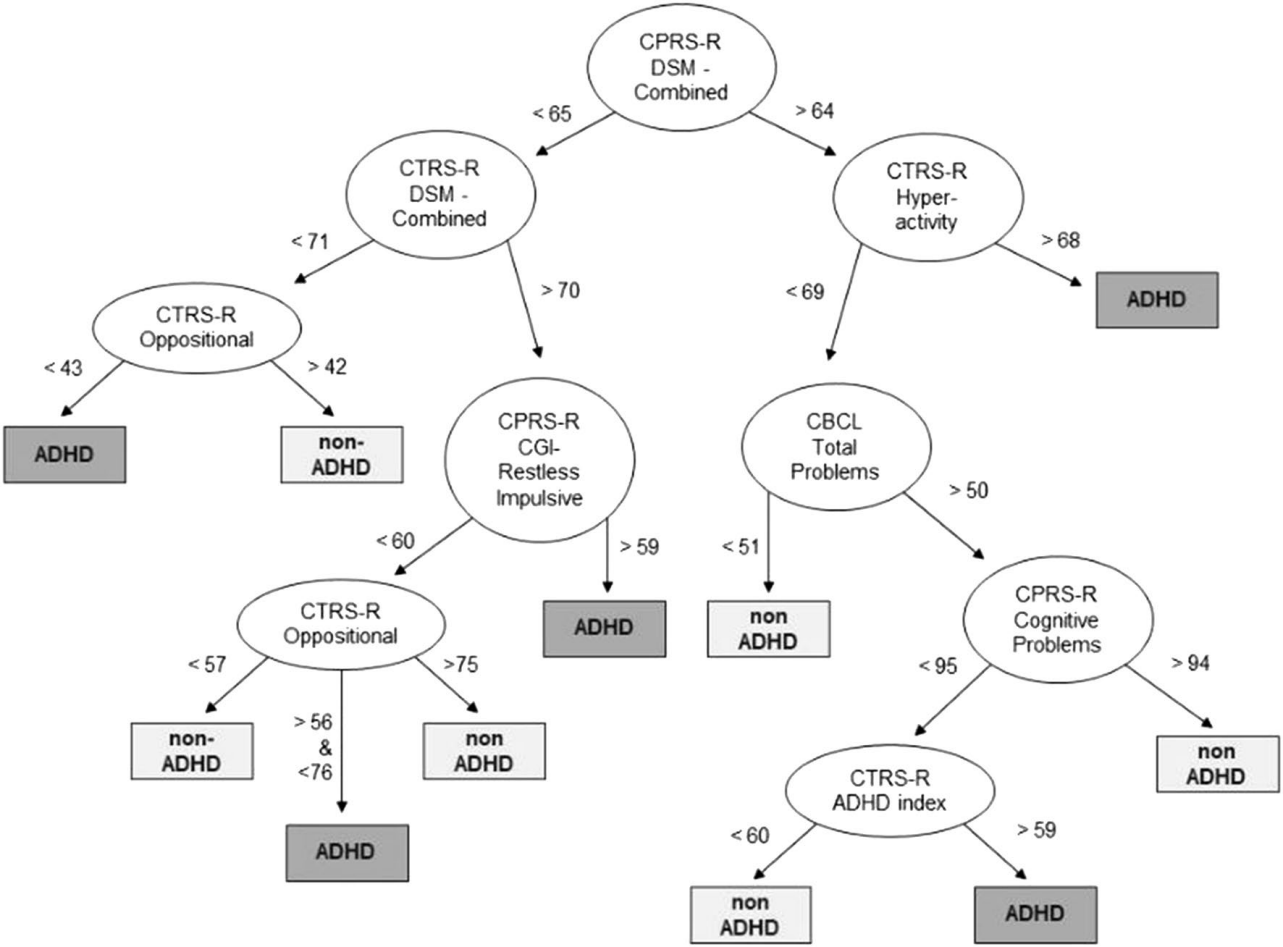
ADHD decision tree results. Representation of the machine learning algorithm results. *ADHD* Attention Deficit and Hyperactivity Disorder, *CBCL* Child Behavior Checklist/ 6–18, *CPRS-R* Conners' Parent Rating Scale-Revised, *CTRS-R* Conners' Teacher Rating Scale-Revised

Figure 2 shows the DT model built considering the whole data set as a training set. This model proved accuracy in 82% of the sample’s subjects.

### Cross-validation experiment of DT model and its predictive accuracy in comparison to other ML models

Table 2 depicts the results of the cross-validation experiment on the DT model and its predictive performance compared to other ML models.

**Table 2:** Classification of ADHD vs non-ADHD subjects: ML models performances on the test sets

Model	Accuracy [95% C.I.]	NIR (p-value)	Specificity	Sensitivity	PPV	NPV
DT	74% [69%–79%]	59% (*p* < 0.001)	50%	92%	72%	80%
RF	74% [69%–79%]	59% (*p* < 0.001)	67%	79%	77%	69%
SVM	75% [70%–80%]	59% (*p* < 0.001)	66%	81%	77%	71%

*C.I.* Confidence interval, *DT* Decision tree, *NIR* No information rate, *NPV* Negative predictive value, *PPV* Positive predictive value, *RF* Random forest, *SVM* Support vector machine

### Identification of DT rules for correct/incorrect classifications

Correct (TP and TN) and incorrect (FP and FN) classifications were considered in the two groups of clinician-diagnosed ADHD and non-ADHD, separately.

**Fig. 3:**
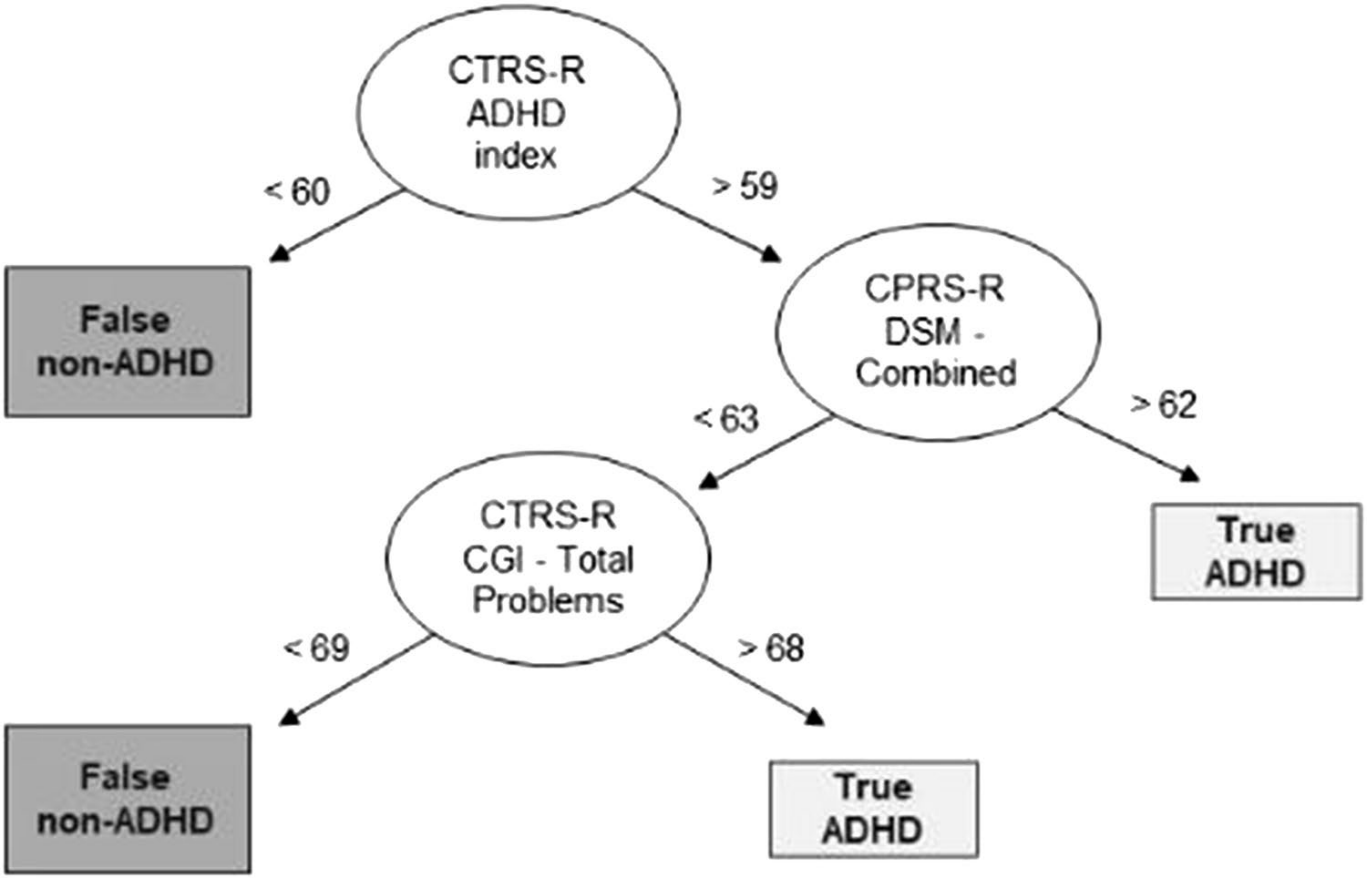
Decision tree results for TP and FN. Representation of the machine learning algorithm results. *ADHD* Attention Deficit and Hyperactivity Disorder, *CBCL* Child Behavior Checklist/ 6–18, *CPRS-R* Conners' Parent Rating Scale-Revised, *CTRS-R* Conners' Teacher Rating Scale-Revised

**Fig. 4:**
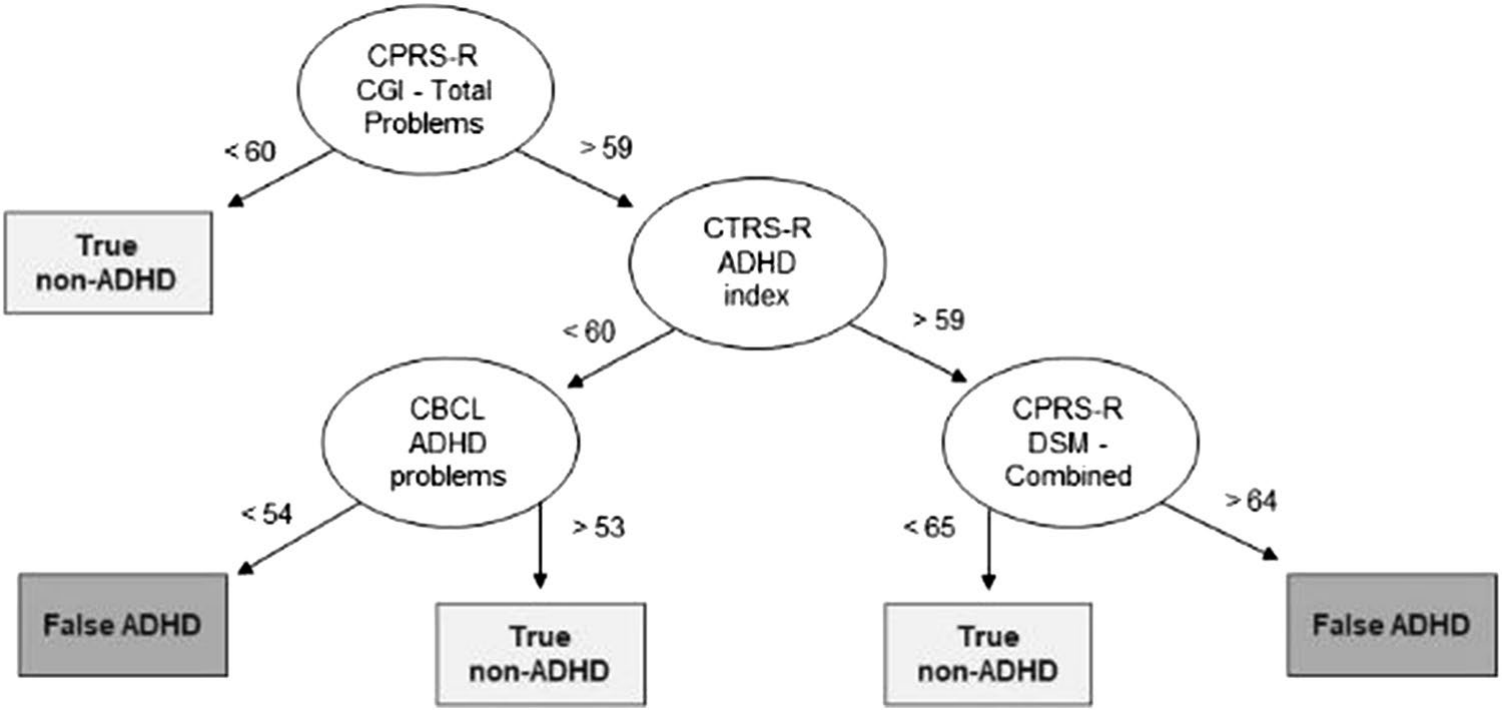
Decision tree results for TN and FP. Representation of the machine learning algorithm results. *ADHD* Attention Deficit and Hyperactivity Disorder, *CBCL* Child Behavior Checklist/ 6–18, *CPRS-R* Conners' Parent Rating Scale-Revised, *CTRS-R* Conners' Teacher Rating Scale-Revised

Figures 3 and 4 show the DT models built considering all the TP/FN subjects and all the TN/FP subjects, respectively.

Tables A1 and A2 in the Supplementary Information section depict the ML models’ predictive performance.

Table 3 shows a rule-based interpretation of the results presented in Figs. 3 and 4.

**Table 3:** Rules for incorrect decision tree-performed classifications

FN class	If CTRS-R ADHD index < 60, or If CTRS-R ADHD index > 59, and CPRS-R DSM—Combined < 63, and CTRS-R CGI—Total < 69
FP class	CPRS-R CGI—Total > 59, and CTRS-R ADHD index < 60, and CBCL—ADHD Problems < 54, or CPRS-R CGI—Total > 59, and CTRS-R ADHD index > 59, and CPRS-R—DSM Combined > 64

Lastly, socio-demographic and cognitive information did not result significantly different between the DT-identified classes.

### Analysis of autism symptoms in correctly/incorrectly classified ADHD children

Table 4 shows the significant results on Fisher’s exact test.

**Table 4:** ADHD and ASD contingency table

	Misclassified as non-ADHD	Correctly classified as ADHD
Absence of ASD	18%	82%
Presence of ASD	0%	100%

Fisher’s exact test addressed the association between categorical ASD diagnosis and FN/TP. *p*-value = 0.029. *ADHD *Attention defici/hyperactivity disorder, *ASD *Autism spectrum disorder

The Kruskal–Wallis tests by ranks were all significant, except for the Social Motivation problems scale. Autism symptoms were reported higher in the TP group compared to the FN group, and in the FP group higher compared to the TN group. Hence, the DT algorithm highlighted a tendency toward an ADHD diagnosis when parents reported elevated ASD symptoms.

**Fig. 5:**
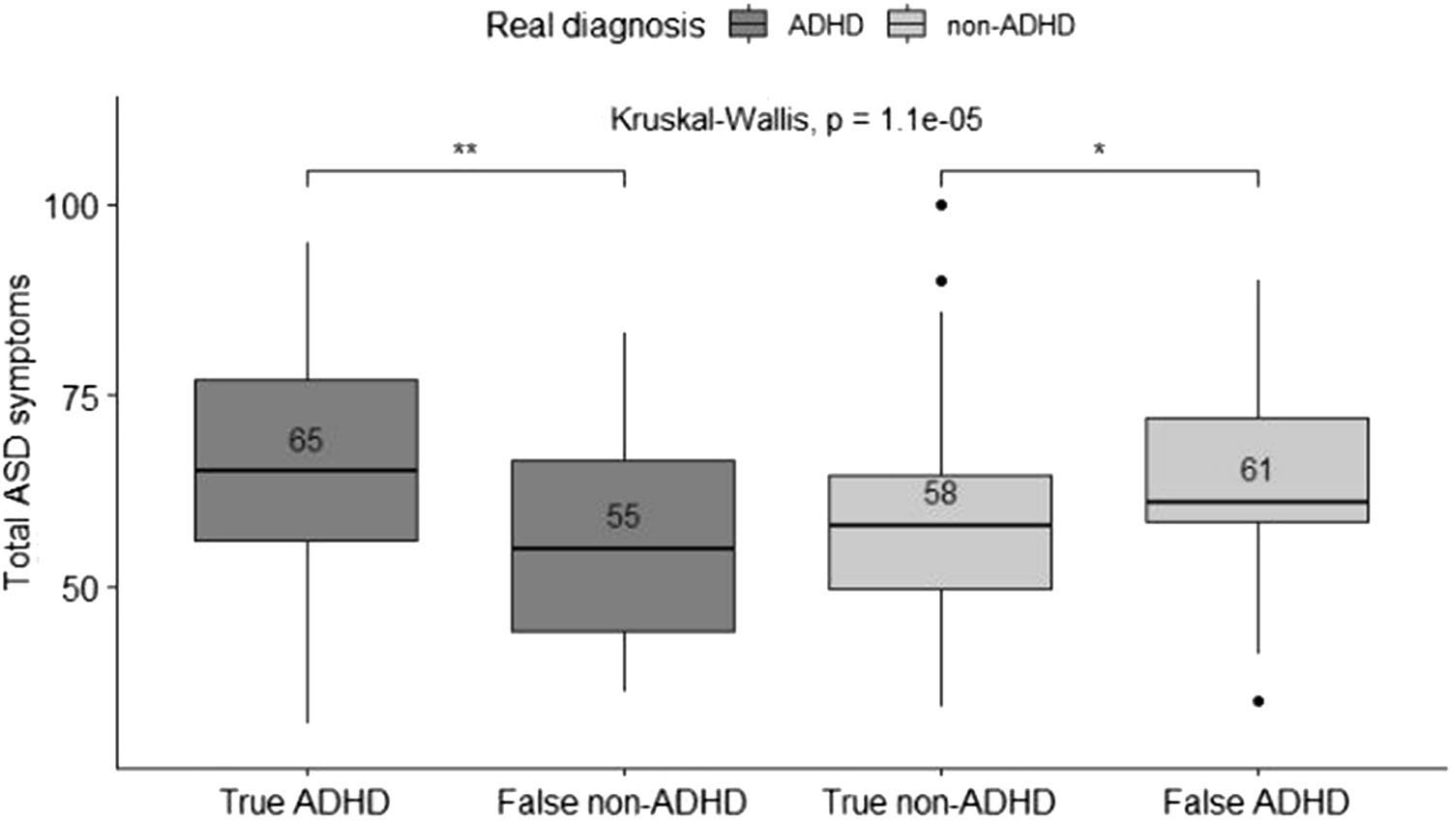
Total ASD symptoms. Differences in total ASD symptoms measured through SRS in the four classes. Note: median values are shown in the boxplots. *p < 0.05, **p < 0.01

Figures A1–A5 in the Supplementary Information section show the results regarding the SRS subscales. As an example, Fig. 5 shows the total score results.

## Discussion

Over the last few years, the digital innovation process and the COVID-19 pandemic have spurred an increasing request for telehealth procedures [37, 38]. The first steps of the diagnostic process for ADHD may fit this trend because a thorough information collection regarding children’s behaviors potentially could be performed remotely [11].

The present study’s first aim was to explore whether and to what extent expert clinicians’ clinical diagnosis of ADHD agreed with symptoms that parents and teachers rated through online-administered questionnaires. To this end, we tested a DT, given its notable interpretability and the suitability for digitally collected data [12]. Our algorithm reached a very good accuracy rate (82%) in correctly identifying children in the training set, which either did or did not receive a diagnosis of ADHD at the end of the clinical evaluation. The present training accuracy is in line with previous ML works that highlighted the possibility of accurately discriminating subjects with and without ADHD [13, 35, 39]. However, earlier research was based on biological, neurophysiological, or behavioral data collected on-site. Thus, this study provided first preliminary evidence, within an Italian setting, that data collected through telehealth might be valuable to support the clinical practice of diagnosing ADHD. Indeed, although no automatic algorithm can substitute clinicians in diagnostic decisions, the proposed DT represents an innovative computational tool that could aid the diagnostic process of ADHD given its automated and interpretable design. Our DT model also reached an accuracy rate of 74% after the cross validation, indicating a good level of generalizability of the present preliminary findings. Lastly, DT algorithm’s performance was directly comparable to those achieved by other less readily interpretable ML models, such as RF and SVM, which achieved an accuracy rate of 74% and 75%, respectively.

As expected, among all the collected measures, the core parent- and teacher-reported ADHD symptom severity was the most discriminative information for the DT classification. Ratings on *DSM*-oriented ADHD scales of both the informants showed a crucial relevance for the clinicians’ diagnostic decision. This is interesting considering that the DT assigns the same “weight” to all the considered input variables (i.e., case history, demographic, cognitive, behavioral). Moreover, although the algorithm was totally naïve about the questionnaire cutoffs, in the upper nodes, the DT identified scores that were in line with moderate and severe risk for ADHD, respectively 64 and 70 [19, 24]. These findings thus extend—for the first time in a telehealth setting—recent findings that showed caregivers’ reports could reliably predict an ADHD diagnosis [8].

Notably, in 18% of the cases, clinicians reached a different diagnostic conclusion compared to that resulting from the parent report-based algorithm. This result is in line with Tahıllıoğlu and colleagues’ (2021) recent findings, which reported that, in 16% of the cases, parent reports were not in line with the diagnostic conclusion clinicians made on the presence or absence of ADHD in a sample of boys [8]. In our sample, the participants incorrectly classified through the DT model did not significantly differ in socio-demographic and cognitive characteristics from those who were correctly classified. However, they presented “extreme” scores on caregivers’ reports, that is, their parents rated their behavioral/emotional difficulties either as significantly higher or significantly lower than those of participants the DT algorithm correctly classified. For those cases, the clinicians’ decisions relied mostly on their direct observation of the patient, on cognitive performance, and on clinical interviews.

The second aim of this study was to understand whether the co-presence of ASD symptoms as the caregivers described could represent a potential confounding factor, given the considerable overlap in symptom presentation [40]. In our sample, 12% of children diagnosed with ADHD were also clinically diagnosed with ASD. This is in line with recent evidence [41].

It is important to remember that the DT only relied on caregivers’ reports of ADHD core symptoms and often associated oppositional symptoms [42]. As expected, the algorithm did not select ASD symptoms as discriminant information for a correct ADHD classification. Nevertheless, all participants clinically diagnosed with comorbid ADHD–ASD the DT algorithm correctly recognized as ADHD; conversely, not all participants with a clinical diagnosis of ADHD without an ASD diagnosis were correctly classified as ADHD. Therefore, this showed that both parents and teachers provided more severe ratings of ADHD in children the clinicians diagnosed with a comorbid ADHD–ASD. The present finding is in line with a recent review, which reported higher externalizing difficulties in children with ASD [40] and with previous evidence describing an additive effect of symptom severity in children with an ADHD/ASD comorbid state [43, 44].

Consistently, the analysis of social abilities among the four groups the DT sorted showed that participants with higher ratings of social cognition, communication problems, and autistic mannerisms on SRS were classified as having ADHD, leading to many FPs for the algorithm. These traits, with the addition of social awareness, were conversely lower in children the algorithm misclassified as non-ADHD. Despite representing a novelty for what concerns the analytic approach, this finding may corroborate previous evidence. Indeed, social functioning atypicalities, a hallmark of ASD, are often reported in ADHD as well [44]. Although research suggests that the mechanisms underlying these difficulties differ [44], social impairment in the two conditions may look similar on a phenotypic level for both clinicians and non-clinicians. Hence, parents of children referred for suspected ADHD may report impaired social functioning, influencing the DT results. An interesting exception to this trend is social motivation scores on SRS. Indeed, all four classes presented typical levels of social motivation, which is in line with a recent work reporting comparable scores in social motivation between children with ADHD only and neurotypical peers [45]. To our knowledge, the present study addressed for the first time ASD features’ effects on an ML algorithm classification of ADHD. Thus, although this opens up possibilities for digitalized support to diagnostic decisions against the background of recent developments in computational psychometry applied to the evidence-based psychological assessment of ADHD [46], the confounding effect of non-core associated symptoms needs to be further investigated in future studies.

### Conclusion

Online information collection and screening procedures should not be merely considered as an alternative to on-site diagnostic practice. Instead, telehealth can help effectively collect reliable caregivers’ reports and obtain a subsequent automated output regarding a diagnostic risk factor [11]. Special attention should be given not only to developing accurate diagnostic classification models but also to the factors that might lead to diagnostic misclassification. Lastly, if the first diagnostic steps are optimized, the time delay between initial symptom detection and diagnosis could reduce, enabling clinicians to focus on the most complex cases.

### Limitations

Some limitations of the present preliminary study should be considered. Our sample included children and adolescents from the same area (Northern Italy) that their pediatricians referred for suspected ADHD. Moreover, the sample is composed of males in a greater proportion compared to females; despite this being in line with literature, it could have represented a bias within our statistical models. Thus, our results might not be generalized to different populations. Furthermore, our analysis exclusively addressed the potentially confounding effects of autism symptoms in ADHD classification. However, there are several conditions commonly associated with ADHD [47]. Additional research addressing these symptoms and conditions’ effects in predicting ADHD is needed. Future research could replicate this line of analyses through a prospective structure because the retrospective approach could have represented a potential bias in our models.

### Future directions

Future research focused on developing online platforms for remotely performed data collection is needed [11]. Developments of ML predictive models could also offer clinicians prompt feedback about the diagnostic risks associated with questionnaire scores. If proven valid, these procedures could be readily implemented for other neurodevelopmental conditions as well.

## Supplementary Information

Below is the link to the electronic supplementary material.

Supplementary file1 (DOCX 47 KB)

## Data Availability

The training data presented in this study are available from the corresponding author upon reasonable request.
