# Supplementary material for: Exploring telediagnostic procedures in child neuropsychiatry: addressing ADHD diagnosis and autism symptoms through supervised machine learning
Source: Eur Child Adolesc Psychiatry. 2023 Jan 25;33(1):139–49. doi: 10.1007/s00787-023-02145-4 (PMC9875192; doi:10.1007/s00787-023-02145-4)
Supplement: Supplementary file 1 — Supplementary file1 (DOCX 47 KB) [file 787_2023_2145_MOESM1_ESM.docx]

**Data cleaning**

Data underwent three stages before they were judged fit for statistical analyses, following the steps depicted by De Jonge and Van Der Loo, 2013 [43]. At each step, raw data and the obtained data frame were saved separately to provide a reproducible flow of data manipulations.

*From raw data to technically correct data*. Clinicians provided an .xls data frame containing raw data from the patients’ clinical records. However, in raw data frames columns may present inconsistencies and characters may be expressed in various formats such as upper and lower case, presenting white spaces, and the same categories may be indicated through different strings. These possible irregularities were addressed, and thus technically correct data were obtained.

*From technically correct data to consistent data*. Technically correct data present values clearly recognizable as belonging to a certain variable and stored as a data type that correctly represents the value domain of that variable. However, technically correct data may still present missing values, outliers, and obvious errors. These were addressed by applying to each variable the summary()R function, which provides basic descriptive information; obvious errors were hence manually fixed. Missing values were addressed as follows: first, the percentage of empty cells was computed for each subject, separately. Subjects presenting 50% or more of missing data were then excluded from the following analysis since these statistical units were not considered informative enough. Empty cells referred to subjects with less than 50% of missing data were then filled through a hot deck imputation [44], after stratifying the dataset by presence or absence of ADHD diagnosis. Lastly, outliers from quantitative variables were detected through the boxplot.stats() R function; however, these values were not removed since they could be representative of the clinical specificities. As a result, a consistent data frame was obtained.

*Consistent data frame*. A consistent data frame is in line with the real-world evidence about the phenomenon described by the data. The data frame obtained through the above-mentioned procedure was considered in the following analyses.

Raw data consisted in a 342x50 .xlsx table containing raw information on children and adolescents evaluated for suspected ADHD through parent and teacher reports. To obtain a technically correct data frame, the dataset was imported in R and ordered by subjects’ identification code, with string variables converted into factors. Moreover, 16 statistical units that presented more than 49% of missing information were removed from the data frame. The maximum percentage of missing information per column was 10%, with reference to the SRS questionnaire; the missing values were filled-in through a hot-deck imputation, with stratification by ADHD diagnosis. Hence, a consistent 326x50 R data frame was obtained.

**Multicollinearity**

Before performing the following statistical analyses, Spearman's linear correlation coefficients were calculated considering all the possible quantitative attribute pairs. Indeed, the variable number was high and the presence of multicollinearity between predictors may affect the model results [45]. Hence, to obtain a dimensionality reduction, one of the two attributes linked by a linear correlation coefficient ≥ 0.8 was removed from the following analyses [46]. Specifically, the following variables were removed to reduce multicollinearity: CBCL Withdrawn/Depressed and ADHD problems; SRS Communication and Autistic Mannerisms; CTRS-R CGI - Total.

**Identification of DT rules for correct/incorrect classifications:**

**predictive performance of the ML models**

| **Table A1 - Classification of TP vs FN: ML models performances on the test sets**. Note: C.I. = Confidence interval; DT = Decision tree; FN: False negative; NIR = No information rate; NPV = Negative predictive value; PPV = Positive predictive value; RF = Random forest; TP = True positive. | | | | | | |
| --- | --- | --- | --- | --- | --- | --- |
| **Model** | **Accuracy [95% C.I.]** | **NIR (p-value)** | **Specificity** | **Sensitivity** | **PPV** | **NPV** |
| DT | 96% [93%-99%] | 84% (p < 0.001) | 98% | 96% | 99% | 83% |
| RF | 97% [93%-99%] | 84% (p < 0.001) | 100% | 96% | 99% | 83% |
| SVM | 100% [98%-100%] | 84% (p < 0.001) | 100% | 100% | 100% | 100% |

| **Table A2 - Classification of TN vs FP: ML models performances on the test sets**. Note: C.I. = Confidence interval; DT = Decision tree; FP: False positive; NIR = No information rate; NPV = Negative predictive value; PPV = Positive predictive value; RF = Random forest; TN = True negative. | | | | | | |
| --- | --- | --- | --- | --- | --- | --- |
| **Model** | **Accuracy [95% C.I.]** | **NIR (p-value)** | **Specificity** | **Sensitivity** | **PPV** | **NPV** |
| DT | 95% [90%-99%] | 80% (p < 0.001) | 89% | 96% | 97% | 86% |
| RF | 95% [90%-98%] | 80% (p < 0.001) | 100% | 94% | 79% | 100% |
| SVM | 100% [97%-100%] | 80% (p < 0.001) | 100% | 100% | 100% | 100% |

**Analysis of autism symptoms in correctly / incorrectly classified ADHD children**


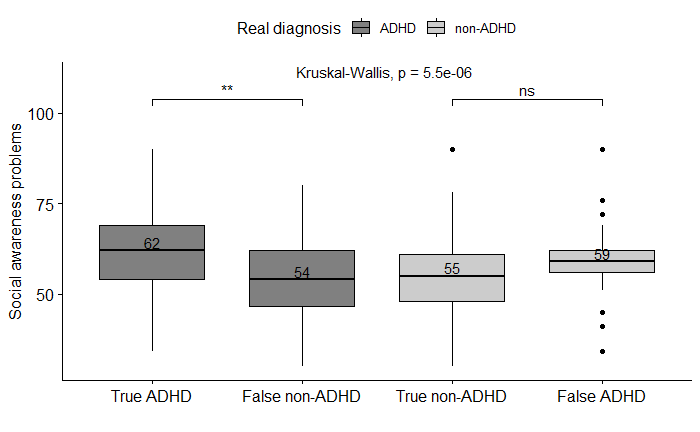


Figure A1: Differences in social awareness problems measured through SRS in the four classes. Note: median values are shown in the boxplots. ** p < 0.01; ns = non-significant.


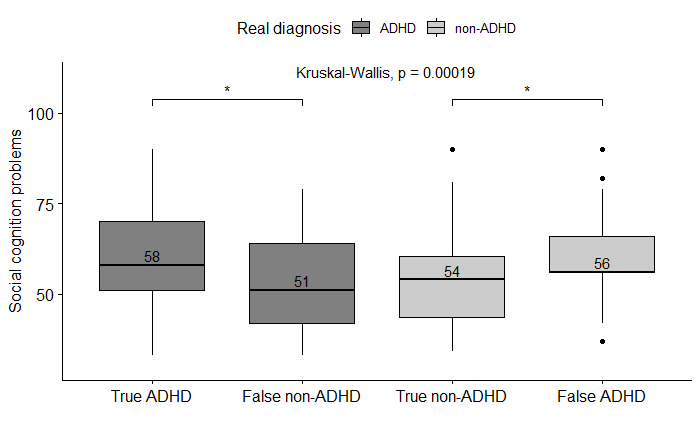


Figure A2: Differences in social cognition problems measured through SRS in the four classes. Note: median values are shown in the boxplots. * p < 0.05.


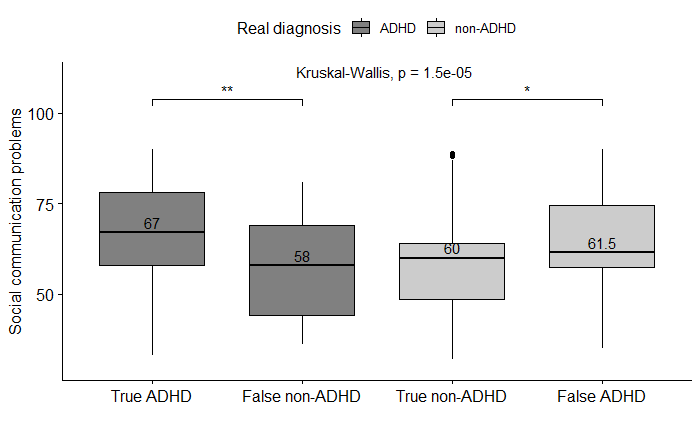


Figure A3: Differences in social communication problems measured through SRS in the four classes. Note: median values are shown in the boxplots. * p < 0.05; ** p < 0.01.

**
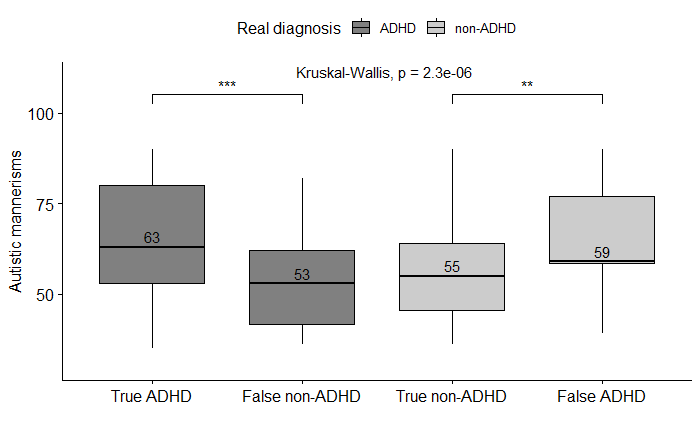
**

Figure A4: Differences in autistic mannerisms measured through SRS in the four classes. Note: median values are shown in the boxplots. ** p < 0.01; *** p < 0.001.

**
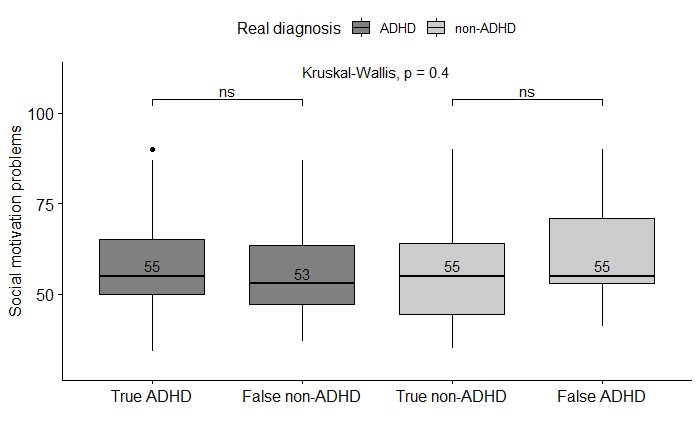
**

Figure A5: Differences in social motivation problems measured through SRS in the four classes. Note: median values are shown in the boxplots. ns = non-significant.
